# The Fatigue Wear Process of Rubber-Metal Shock Absorbers

**DOI:** 10.3390/polym14061186

**Published:** 2022-03-16

**Authors:** Marcin Kluczyk, Andrzej Grządziela, Michał Pająk, Łukasz Muślewski, Adam Szeleziński

**Affiliations:** 1 Mechanical and Electrical Engineering Faculty, Polish Naval Academy in Gdynia, 81-127 Gdynia, Poland; a.grzadziela@amw.gdynia.pl; 2 Mechanical Engineering Faculty, University of Technology and Humanities in Radom, 26-600 Radom, Poland; m.pajak@uthrad.pl; 3 Mechanical Engineering Faculty, University of Science and Technology in Bydgoszcz, 85-796 Bydgoszcz, Poland; lukasz.muslewski@utp.edu.pl; 4 Department of Maritime Engineering, Gdynia Maritime University, 81-225 Gdynia, Poland; a.szelezinski@wm.umg.edu.pl

**Keywords:** marine absorber, rubber, fuel and oil ageing, vibrations

## Abstract

Rubber and rubber-metal vibration isolators are widely used vibration isolation systems in marine applications. For naval application, shock absorber mounting systems must fulfil two functions. The first one supports the suspended mass in the absence of waving or detonation while providing isolation from vibrations and shock impact. In the second case, during the machine operation, it reduces the force of movement to an acceptable value. Moreover, it returns the insulated mass to the position output without plastic deformation or residual buckling after removing shock stresses or harmonic vibrations. The environment in which marine vibration isolators are to be used strongly influences the selection of a shock absorber. The main environmental problem is the temperature range in marine power plants, which ranges from 20 °C to 55 °C. Temperature fluctuations may cause changes in the physical properties of typical vibration/shock insulators. Both rubbers and elastomers used for shock absorbers tend to stiffen, gain low-temperature damping, and soften and lose damping at elevated temperatures. Factors such as moisture, ozone and changes in atmospheric pressure are usually ignored in shipbuilding. The main environmental factors influencing the ageing of insulators are liquid saturated hydrocarbons, i.e., oils, fuels, coolants, etc., which may come into contact with the surface of the insulators. This work presents the results of the research carried out to determine the effect of overload and the impact of petroleum products on the materials of metal-rubber shock absorbers made of three different rubbers and one polyurethane mixture. For each of the materials, shock absorbers with three different degrees of hardness were tested.

## 1. Introduction

Elastomer insulators are the most commonly used type of vibration dampers. The elastomer is natural rubber or any polymer with elastic properties similar to natural rubber. Such materials are widely used in insulators because they can be easily formed into any desired shape and selected to provide a wide range of stiffnesses. Such insulators have better damping properties than metal springs, usually require less space, are lightweight and can be combined with metal inserts for simple assembly [1].

Elastomer insulators are able to withstand high deformation and then return to their original state with virtually no damage or change of shape. Additionally, they are superior to other types of insulators in terms of the given resilience and ability to deflect and dissipate energy. They require less space and weight. Elastomers are characterized by exceptional extensibility and deformation, can be used with elongations up to about 300%, and the final elongation of some elastomers can reach 1000%. They can be stressed up to 10 MPa or more before reaching the yield point [2,3,4]. Their excellent ability to store elastic energy enables the transfer of considerable stresses, thanks to which, after being released from stresses, they practically return to their original shape without damage. Elastomer damping is often useful in preventing excessive vibration amplitude in resonance.

The use of elastomeric materials to construct vibration isolators and shock absorbers results from the specific physical properties of high strength, high fatigue resistance, moderate production costs, excellent rebound and compression properties. Determining the properties of the material and its structure, as well as the installation method in an insulated machine, it is necessary to assess the effectiveness of the shock absorber application [1,5,6]. Marine vibration isolators and shock absorbers used in naval vessels are mainly used to ensure three technological parameters, i.e., maintaining the alignment of rotary machines, isolating harmonic vibrations and absorbing energy at shock impacts. It is extremely difficult to meet these requirements mainly due to the significantly different dynamics of these phenomena. Normal operation of the propulsion system causes the formation of harmonic vibrations, while the impact of the shock wave from an underwater explosion is an impulse feature.

The analysis of the used rubber and rubber-metal shock absorbers most often concerns three issues, i.e., cyclical changes in properties, reaction to large deformations and non-linearity of the stress–strain curve. In the case of materials used for shock absorbers, these parameters have been theoretically characterized and practically verified [7,8,9,10,11]. The durability of shock absorbers is of fundamental importance in shipbuilding engineering, which is mainly related to the resistance to cyclical loads and environmental impacts, primarily related to temperature and immersion in aggressive liquids [9,10,11,12,13,14,15]. According to [16,17] one of the most widely used techniques to predict the lifetime of polymeric materials is the use of the Arrhenius equation; however, it will not be used in this work. 

Shock absorbers that are subjected to cyclical loads often fail due to the breakage of the rubber element [18,19,20]. A crack can be initiated in the area where the stress concentration is the maximum of the probability of cracking increases with the service life. Dimensional stability is necessary for vibration isolators that operate under applied loads, which means that the static deflection of the insulator should not increase with time. Such an increase would be the result of creep and deformation. Strain is a dimensional change due to stress, and creep is a dimensional change due to the application of a force. The deformation and excessive creep will cause large changes in stiffness and dynamic properties. Deformation is determined by compressing a specimen of specified dimensions to a given deflection [7]. After compression is complete, the sample is left for half an hour and then subjected to an elevated temperature. After exposure, the sample is left for half an hour, and then the thickness is measured. The compression deformation percentage is the thickness decrease divided by the original deformation and multiplied by 100. Typical rubber compounds used in vibration insulation have a value of 10 to 50%. The exposure time is typically 22 or 70 h at a temperature suitable for the intended use of the insulator or silencer. Creep is determined by placing the sample in a compression device, applying a compressive force, and exposing it to an elevated temperature.

Some vibration isolators come into contact with oil or solvents. The influence of these liquids on a given rubber depends on the solubility parameters of both materials. The greater the similarity, the greater the effect. The liquid may cause the rubber to swell and may absorb chemicals from it or react chemically with it. Each of these factors can lead to deterioration of the physical properties of the rubber. The effect of liquids on rubber is determined by measuring changes in volume or weight, hardness, elongation and tensile strength after immersion in oils, fuels, operating fluids or water [4,8,10,11,21,22]. Many research centers around the world develop guidelines for standardized tests of all kinds of rubber elements. Institutions such as the American Society for Testing and Materials (ASTM) or the International Organization for Standardization (ISO) publish standards covering procedures for testing rubber elements for environmental impact [5,8,23]. However, there is little information in the literature known to the authors about changes in the characteristics of metal-rubber vibro-isolators in the event of a significant overload. The same applies to changes in the resonance characteristics in the case of the impact of petroleum products on them. Therefore, the authors decided to take up the topic presented in this study.

## 2. Materials and Methods

**Figure 1:**
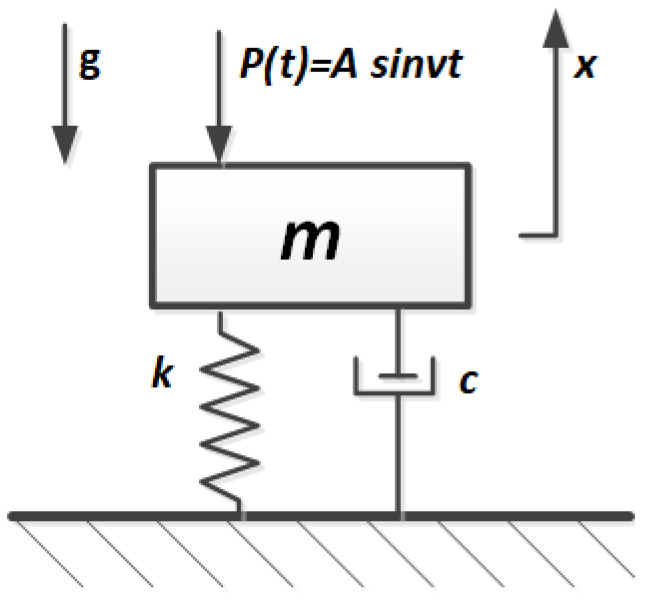
System of the shock absorber.

This work focuses on determining the effects of overload and oil immersion interactions, which are most quickly visible in the form of stress relaxation and creep and assessing the possibility of the dramatic Mullins effect, which may disqualify the shock absorber from use. Contact or immersion in aggressive liquids also affects the properties of elastomeric materials. This effect should be related to the determination of changes in the natural frequency due to changes in stiffness and damping [6,9,21]. By analyzing the work of a shock absorber in good condition (Figure 1), it can be described by the commonly known equation of forced vibration with damping [23]:

(1) $$m\ddot{x}+c\dot{x}+kx=A\sin\upsilon t$$

where m—mass, c—damping, k—stiffness, x—displacement, $P_{a}$—force, ω—angular frequency, t—time, *P*(*t*) = *A*sin*vt*—exciting force of amplitude *A* and frequency *v*.

After dividing by *m*, we obtain [23]:

(2) $$\ddot{x}+2h\dot{x}+\omega_{0}^{2}x=q\sin\upsilon t$$

where

(3) $$h=\frac{c}{2m};\;\;\;q=\frac{A}{m};\;\;\omega_{0}=\sqrt{\frac{k}{m}}$$

The solution to Equation (2) is a superposition of two solutions to equations, the ordinary and the specific:

(4) $$x=x_{0}+x_{s}$$

The general solution takes the form of:

(5) $$x_{0}=e^{-ht}\left(C_{1}\cos pt+C_{2}\sin pt\right)$$

A specific solution will take the form of:

(6) $$x_{s}=C_{3}\sin\upsilon t+C_{4}\cos\upsilon t$$

Individual derivatives have the form:

(7) $$\dot{x_{s}}=C_{3}\upsilon \cos\upsilon t-C_{4}\upsilon \sin\upsilon t$$

(8) $$\ddot{x_{s}}=-C_{3}\upsilon^{2}\sin\upsilon t-C_{4}\upsilon^{2}\cos\upsilon t$$

Substituting the relations (5)–(7) into Equation (2), the following was obtained:

(9) $$\begin{array}{c} -C_{3}\upsilon^{2}\sin\upsilon t-C_{4}\upsilon^{2}\cos\upsilon t+2h\upsilon C_{3}\cos\upsilon t-2h\upsilon C_{4}\sin\upsilon t+\\ +\omega_{0}^{2}C_{3}\sin\upsilon t+\omega_{0}^{2}C_{4}\cos\upsilon t-q\sin\upsilon t=0 \end{array}$$

Grouping expressions with cos*vt* and sin*vt* obtained:

(10) $$\left(C_{3}\left(\omega_{0}^{2}-\upsilon^{2}\right)-2h\upsilon C_{4}-q\right)\sin\upsilon t=0$$

(11) $$\left(C_{3}2h\upsilon -\left(\omega_{0}^{2}-\upsilon^{2}\right)C_{4}\right)\cos\upsilon t=0$$

Expressions (9) and (10) will be reset to zero for each t as elements in parentheses will be equal to zero, that is:

(12) $$\left(C_{3}\left(\omega_{0}^{2}-\upsilon^{2}\right)-2h\upsilon C_{4}-q\right)=0$$

(13) $$\left(C_{3}2h\upsilon -\left(\omega_{0}^{2}-\upsilon^{2}\right)C_{4}\right)=0$$

From Equation (12), we determine the quantity *C*_4_.

(14) $$C_{4}=\frac{C_{3}2h\upsilon }{\left(\omega_{0}^{2}-\upsilon^{2}\right)}$$

After substituting Equation (11), the following equation is obtained:

(15) $$C_{3}\left(\omega_{0}^{2}-\upsilon^{2}\right)=\frac{C_{3}4h^{2}\upsilon^{2}}{\left(\omega_{0}^{2}-\upsilon^{2}\right)}-q$$

Hence:

(16) $$C_{3}=\frac{q\left(\omega_{0}^{2}-\upsilon^{2}\right)}{\left(\omega_{0}^{2}-\upsilon^{2}\right)+4h^{2}\upsilon^{2}}$$

Having the expression *C*_3_, the constant *C*_4_ was derived from the dependence (14).

(17) $$C_{4}=\frac{-2h\upsilon q}{\left(\omega_{0}^{2}-\upsilon^{2}\right)+4h^{2}\upsilon^{2}}$$

Thus, the specific solution takes the form:

(18) $$x_{s}=\frac{q\left(\omega_{0}^{2}-\upsilon^{2}\right)}{\left(\omega_{0}^{2}-\upsilon^{2}\right)+4h^{2}\upsilon^{2}}\sin\upsilon t-\frac{2h\upsilon q}{\left(\omega_{0}^{2}-\upsilon^{2}\right)+4h^{2}\upsilon^{2}}\cos\upsilon t$$

Assuming that:

(19) $$\frac{q\left(\omega_{0}^{2}-\upsilon^{2}\right)}{\left(\omega_{0}^{2}-\upsilon^{2}\right)+4h^{2}\upsilon^{2}}=H\cos\varphi$$

(20) $$\frac{-2h\upsilon q}{\left(\omega_{0}^{2}-\upsilon^{2}\right)+4h^{2}\upsilon^{2}}=H\sin\varphi$$

and substituting the expression (18), we obtain:

(21) $$x_{s}=H\cos\varphi \sin\upsilon t+H\;H\sin\varphi \cos\upsilon t=H\sin\left(\upsilon t+\varphi \right)$$

where:

(22) $$\begin{array}{c} H=\sqrt{C_{3}^{2}+C_{4}^{2}}=\sqrt{\frac{q^{2}{\left(\omega_{0}^{2}-\upsilon^{2}\right)}^{2}+4h^{2}\upsilon^{2}q^{2}}{{\left(\left(\omega_{0}^{2}-\upsilon^{2}\right)+4h^{2}\upsilon^{2}\right)}^{2}}}=q\sqrt{\frac{{\left(\omega_{0}^{2}-\upsilon^{2}\right)}^{2}+4h^{2}\upsilon^{2}}{{\left(\left(\omega_{0}^{2}-\upsilon^{2}\right)+4h^{2}\upsilon^{2}\right)}^{2}}}\\ \;\;\;\;\;\;=\frac{q}{\sqrt{\left(\omega_{0}^{2}-\upsilon^{2}\right)+4h^{2}\upsilon^{2}}} \end{array}$$

Then, static extension equals:

(23) $$l_{st}=\frac{A}{k}=\frac{\frac{A}{m}}{\frac{k}{m}}=\frac{q}{\omega_{0}^{2}}$$

The expression (22) can be represented in the form:

(24) $$H=\frac{q}{\omega_{0}^{2}\sqrt{{\left(1-\frac{\upsilon^{2}}{\omega_{0}^{2}}\right)}^{2}+\frac{4h^{2}\upsilon^{2}}{\omega_{0}^{4}}}}=\frac{\frac{q}{\omega_{0}^{2}}}{\sqrt{{\left(1-\frac{\upsilon^{2}}{\omega_{0}^{2}}\right)}^{2}+\frac{4h^{2}\upsilon^{2}}{\omega_{0}^{4}}}}$$

Therefore:

(25) $$x_{s}=\frac{l_{st}}{\sqrt{{\left(1-\frac{\upsilon^{2}}{\omega_{0}^{2}}\right)}^{2}+\frac{4h^{2}\upsilon^{2}}{\omega_{0}^{4}}}}\sin\left(\upsilon t+\varphi \right)$$

Magnification factor is:

(26) $$\mu =\frac{H}{l_{st}}=\frac{l_{st}}{\sqrt{{\left(1-\frac{\upsilon^{2}}{\omega_{0}^{2}}\right)}^{2}+\frac{4h^{2}\upsilon^{2}}{\omega_{0}^{4}}}}\frac{1}{l_{st}}=\frac{1}{\sqrt{{\left(1-\frac{\upsilon^{2}}{\omega_{0}^{2}}\right)}^{2}+\frac{4h^{2}\upsilon^{2}}{\omega_{0}^{4}}}}\;$$

Thus, the complete solution to Equation (4) takes the form:

(27) $$x=x_{0}+x_{s}=e^{-ht}\left(C_{1}\cos pt+C_{2}\sin pt\right)+\frac{\frac{q}{\omega_{0}^{2}}}{\sqrt{{\left(1-\frac{\upsilon^{2}}{\omega_{0}^{2}}\right)}^{2}+\frac{4h^{2}\upsilon^{2}}{\omega_{0}^{4}}}}$$

The constants *C*_1_ and *C*_2_ in the equation must be determined from the assumed initial conditions. The first term of Equation (27) describes the damped vibrations with the frequency *p*, and the second describes the forced vibrations with the frequency of the exciting force *v*. The theoretical analysis of the above equations shows that the change in stiffness caused by contact with an aggressive liquid will cause a difference in the natural frequency $\omega_{n}$ and changes in the stiffness k as well as the amplitude. As a result, changes in the resonance characteristics of the vibrating system can be expected with the time of exposure to an aggressive liquid [22]. 

**Figure 2:**
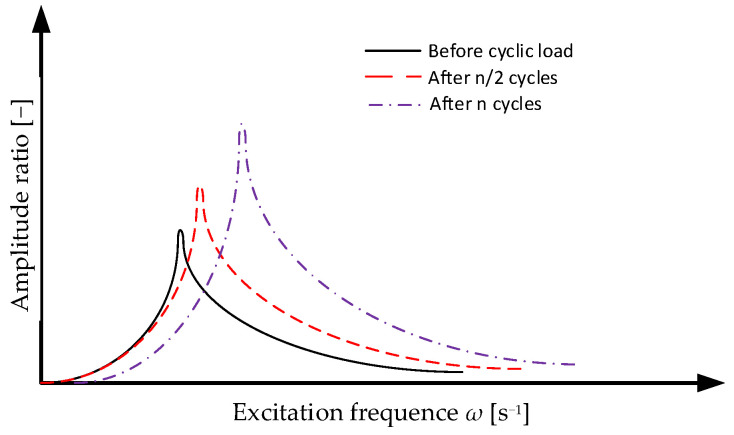
Plots of the dependence of the ratio of amplitudes $x_{a}$*/*$x_{0}$ on the excitation frequency ω for successive, cyclic loads.

Cyclic loads changing the deformation of the shock absorber within the design range of ±20% should be considered as the possibility of changes in the shock absorber geometry due to creep and stress relaxation. Another problem that interested the authors is the change in the stiffness of the shock absorber as a result of cyclic loading (Figure 2). At this stage of the research, the temperature factor was not taken into account as a catalyst for changes in stiffness.

### 2.1. Materials

**Figure 3:**
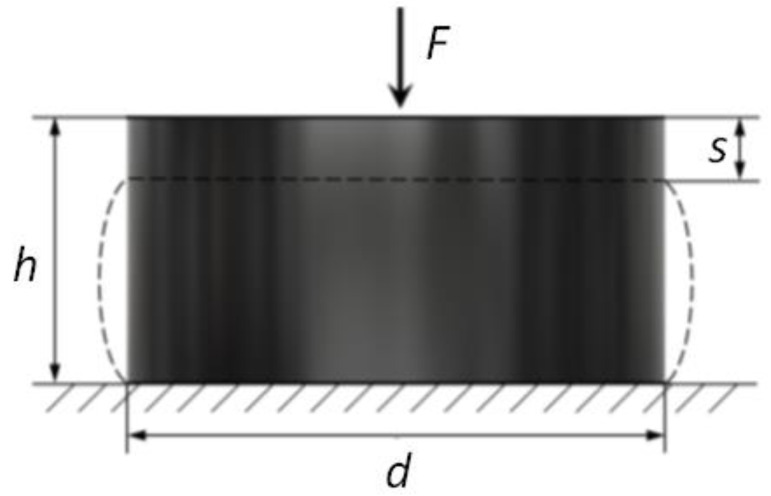
Shock absorber deflection under force F.

Rubber and rubbery-like materials are essentially incompressible substances that deform by changing shape rather than changing volume. Their Poisson’s ratio is around 0.5. At very small deformations, the ratio of the resulting deformation to the applied stress, i.e., Young’s modulus, is constant. This value is the same whether the deformations are the result of tension or compression. Hooke’s law, therefore, applies within the limits of this proportionality. However, as strain increases, this linearity can not be used, and Hooke’s law is no longer applicable. The compressive and tensile stresses are then also different. Typically, rubber vibration dampers are designed to take advantage of these stresses. Compressive stress exhibits a nonlinear cure to strains greater than 30% and is used where restriction of movement is required. Additionally, it is recommended where energy storage is required. Tensile stress stores energy more efficiently than compressive or shear loads, but it is not recommended because of the resulting stresses at the rubber-to-metal interface, which can cause premature failure. The modulus of elasticity is the ratio of stress to deformation expressed in newtons per millimeter [5,23]. It depends not only on the elastic modulus of the rubber but also on the shape of the sample or test piece. Since the rubber is incompressible, compression in one direction stretches in the other two directions, causing the sides to bulge. The shape factor is calculated by dividing one loaded area by the total free area (Figure 3).

For a correct analytical solution of a rubber shock absorber, its shape coefficient k should be determined. This coefficient occurs at the place where the dimension or shape of the loaded elements changes, which is also where the stress distribution changes.

(28) $$k\;=\frac{d}{4h_{s}}$$

During the different test series carried out on the shock absorbers, four types of materials, each with the three most popular levels of hardness in the marine industry—i.e., 55, 65 and 75 Sh A—were tested. 

CR—rubber based on chloroprene rubber. It is one of the first synthetic rubbers. It is distinguished by its above-average resistance to ozone. It is also resistant to weather conditions. It is characterized by high abrasion resistance. Chloroprene rubber is also distinguished by its high resistance to many chemical compounds. However, chloroprene rubber is not resistant to petroleum fuels and oils. Its temperature range of operation ranges from −40 degrees Celsius up to +110 degrees Celsius. For a short time, it can reach up to +130 degrees. Chloroprene rubber is also flexible at temperatures down to about −40 degrees Celsius. However, it is worth remembering that when storing it at a temperature below 0 degrees Celsius during the ongoing crystallization process, an irreversible stiffening process takes place.

EPDM (ethylene propylene diene monomer)—rubber cross-linked in the process of sulfur vulcanization, EPDM has very good properties, including resistance to weather conditions (ozone), water resistance, good high-temperature properties up to +110 °C, flexibility at low temperatures down to −40 °C and hardness ranging from 40 ÷ 90 Shore A. 

NBR (nitrile rubber)—commonly known as oil-resistant rubber, i.e., a copolymer of butadiene and acrylonitrile. Depending on the amounts of the above-mentioned chemical compounds used and the change in their percentage in the mixture, the resistance to oils and low temperature can be freely manipulated. The temperature parameters at which you can work with this rubber range between −30 degrees Celsius and 100 degrees Celsius. The physical properties of rubber are resistance to engine, heating and transformer oils. Rubber is also characterized by high durability to the use of lubricants, hydraulic fluids and aliphatic hydrocarbons [5]. It will respond well to work in conditions with the use of propane, butane and gasoline.

PU—polyurethane is a polymer obtained by polyaddition of aromatic or aliphatic diisocyanates with compounds containing at least two hydroxyl groups. The physical and chemical properties of polyurethanes depend on their composition and molecular weight. Polyurethane elastomers are a rubber-like material with high-performance indicators. Their properties can be shaped in a very wide range, which makes them a universal material. 

### 2.2. Methods

During the research, shock absorbers of identical dimensions, i.e., diameter of 20 mm and height of 40 mm, made of the four different materials described above, were tested. They were subjected to multi-stage strength tests, as well as tests for changes in their dynamic characteristics as a result of ageing in the presence of petroleum products. In the first part of the tests, two series of tests were carried out on a tensile testing machine for each type of shock absorber. Twelve different shock absorbers were tested. 

From each type and hardness, two completely new specimens were randomly selected and tested.

**Figure 4:**
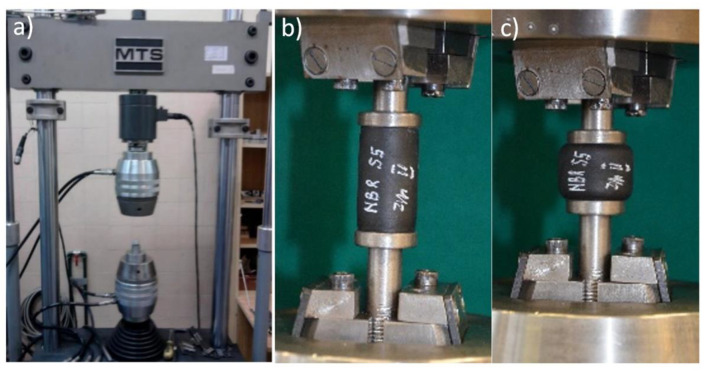
From the left: (**a**)—view of MTS 810.12 tensile machine, (**b**)—NBR 55 absorber during the initial phase o tests, (**c**)—NBR 55 absorber under static compresion of 20 mm.

**Figure 5:**
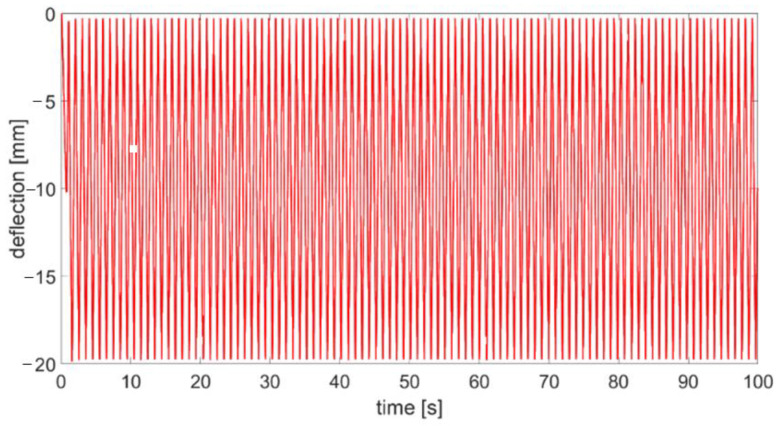
An example of 100 compression cycles at 50% (20 mm) triangular course.

1. For the first samples, the following were carried out:—static compression test for 25% (10 mm)–filename: materialnemestatprzed1; then 10,000 compression cycles at 10% (4 mm), sinusoidal course, finished with static compression test for 25% (10 mm)—filename: materialnamestatpo1.
2. For the second samples of each type, the following were carried out:—static compression test at 25% (10 mm)—filename: materialnamestatprzed2 then 100 compression cycles at 50% (20 mm) triangular course—Figure 4 and Figure 5, finished with static compression test for 25% (10 mm)—filename: materialnamestatpo2.

All compression tests were carried out on the MTS 810.12 (MTS Systems GmbH, Berlin, Germany) machine (Figure 4a), which is a hydraulic testing machine that enables the performance of static tests in the range of +/−90 kN (tensile and compression) for round or flat samples. Machine control enables the carrying out of tests on the basis of a given force, displacement or deformation in a range up to 100 mm. An example course of 100 compressions is presented in Figure 5. Measurement results are archived in ASCII text files. Compresion tests were repeated three times for each type of absorber. An example of a sample (NBR 55) during testing is shown in Figure 4b,c. The tensile machine is certified by the Polish Centre for Accreditation (PCA). The maximum measurement uncertainty for compressive forces is 0.25%.

The test results are presented in the results section. The shock absorbers used for the compression tests on the testing machine were not used for further tests.

**Figure 6:**
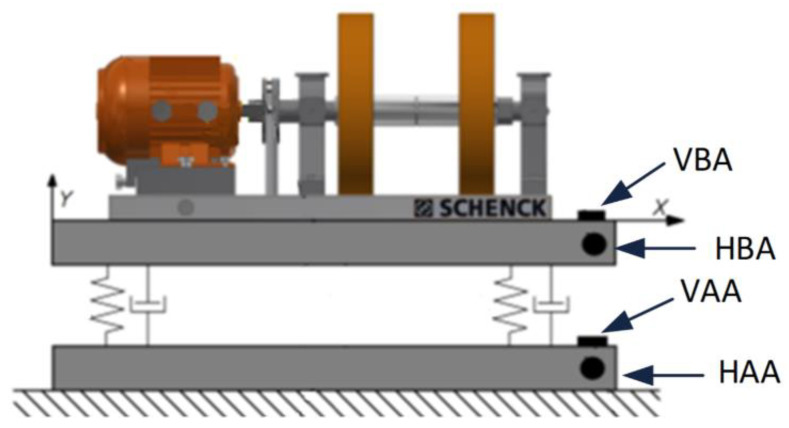
Test stand for dampers long-distance tests. Locations of accelerometers: VBA—vertical before absorber, VAA—vertical after absorber, HBA—horizontal before absorber, HAA—horizontal after absorber.

In the next stage of the study, the tests were carried out in conditions similar to the real ones, i.e., from each group (material-stiffness), four new shock absorbers were selected, which were installed as vibro-isolating elements in the foundation of the machine presented in Figure 6. An artificial unbalance weighing 35 g was introduced in the rotating system of the device. Thanks to this solution, the values of vibration parameters occurring during the operation of the device increased significantly. In the first part of the second stage of the research, new dampers were installed, and the device was turned on. After the rotational speed had stabilized, the vibration acceleration was measured with the use of four accelerometers, the installation locations of which are indicated and described in Figure 6. Then, the power supply to the drive motor was turned off and the vibration parameters were recorded during the free run-down of the rotor system, which allowed for the elimination of disturbances related to the operating electric motor.

After recording the vibration parameters during the run-down of the new shock absorbers, the rotor system was restarted at 500,000 revolutions. In a situation of significant imbalance, it can be assumed that each rotation is the same as a single sinusoidal impulse. The rotational speed of the rotor system is 1500 rpm. After the system had performed the assumed number of revolutions, the measurement was performed again in the steady state and after its completion in the transient state. Due to the duration of registration in individual states, the number of turnovers may differ by 1%. Vibration parameters were measured using the Pulse B&K measuring system (Brüel & Kjær A/S, Virum, Denmark). The apparatus includes four accelerometric sensors type 4514-B from the DeltaTron family, and a six-channel measuring cassette type 3050-A-060 from the LAN-XI series. A high-pass filter was used with a cut-off frequency of 0.7 Hz and the sampling frequency was set to 3092 Hz.

**Figure 7:**
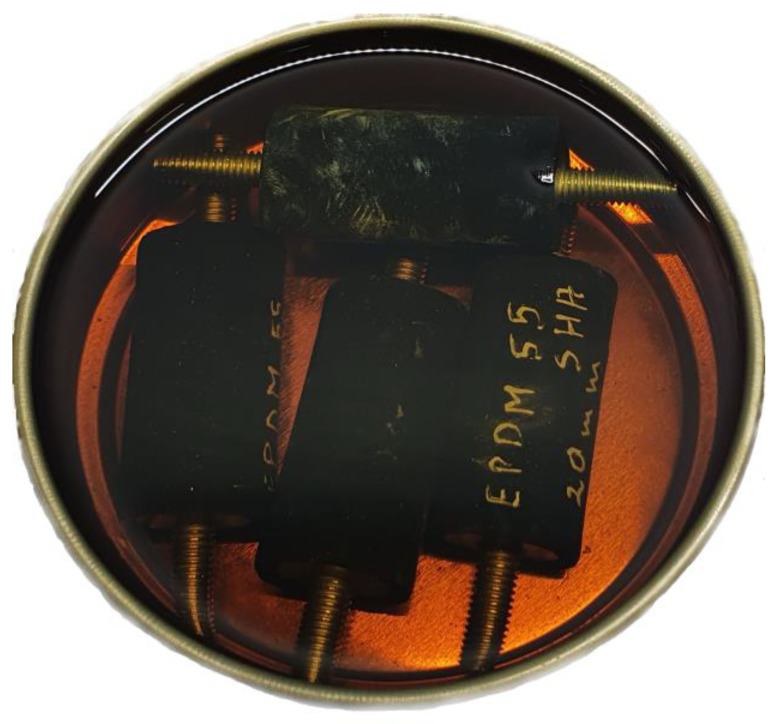
An example of vibroisolators during ageing in fuel and oil mixture.

During the last stage, a completely new set of four shock absorbers from each group (material-stiffness) was subjected to a mixture of petroleum products. Since the tested shock absorbers are used in shipbuilding, a mixture of NATO F75 marine fuel (Lotos, Gdańsk, Poland and Marinol RG1240 marine engine oil (Lotos, Gdańsk, Poland) was prepared as a factor influencing them. The mixture of these two products is a very common factor in ship practice, affecting the rubber elements of the machine foundation system in ship power plants. The mixture was prepared so that 50% of the mass was fuel and 50% was oil. The shock absorbers were immersed in the prepared mixture for 48 h (Figure 7). Before immersion, all shock absorbers were measured and weighed. After aging in the presence of petroleum products, the shock absorbers were dried and re-measured. The results are presented in Table 1. Next, the shock absorbers were mounted in the foundation structure of the machine ilustrated in Figure 6 and subjected to the same tests as in the first part. Thus, measurements were obtained for shock absorbers made of four different materials with three different stiffnesses for the variant of new and aged rubber elements in petroleum products.

## 3. Results 

**Figure 8:**
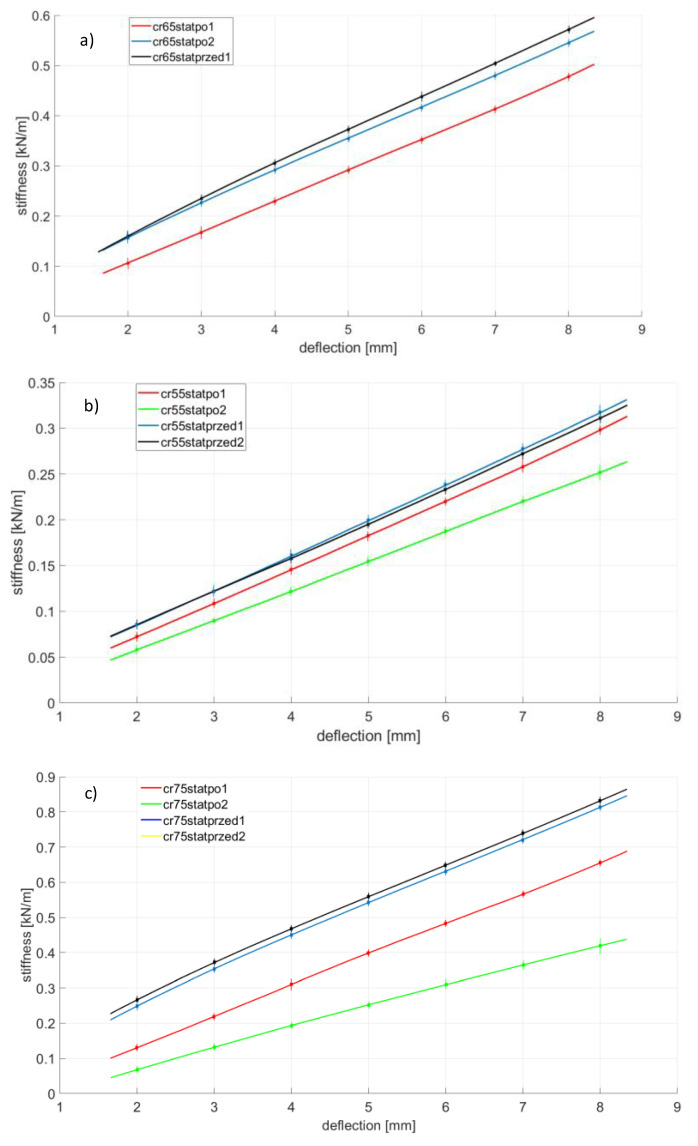
CR rubber changes of stiffness during various tests conducted on tensile machine, (**a**)—CR 55 Sh A, (**b**)—CR 65 Sh A, (**c**)—CR 75 Sh A.

**Figure 9:**
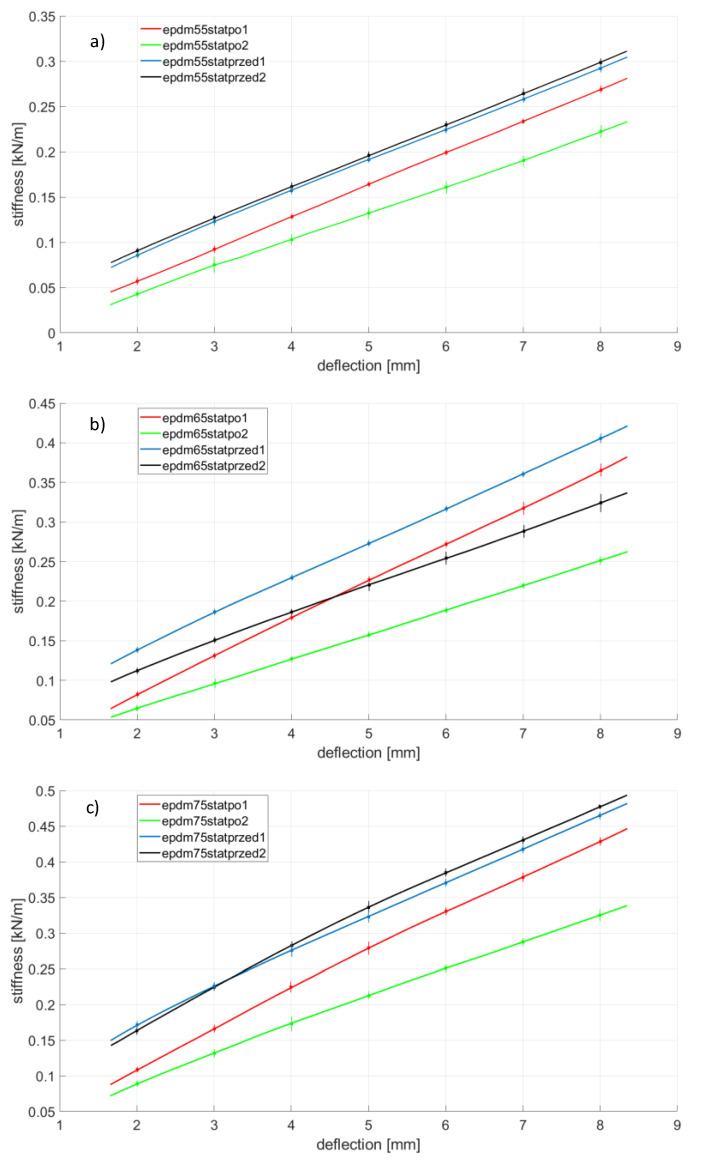
EPDM rubber changes of stiffness during various tests conducted on the tensile machine, (**a**)—EPDM 55 Sh A, (**b**)—EPDM 65 Sh A, (**c**)—EPDM 75 Sh A.

**Figure 10:**
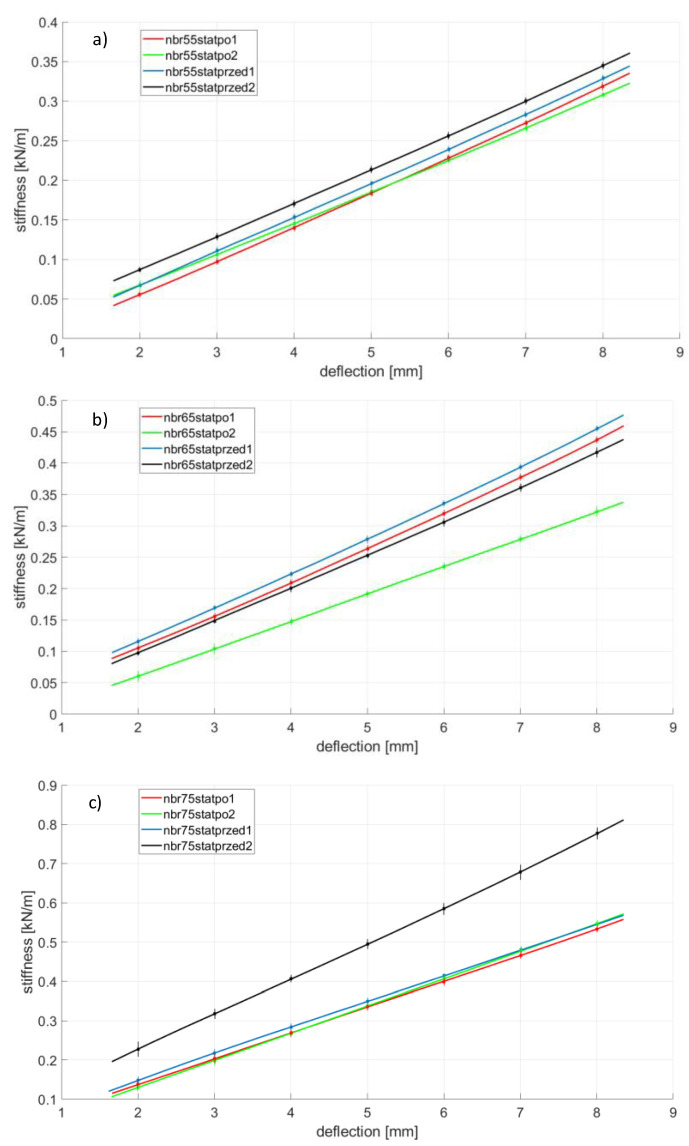
NBR rubber changes of stiffness during various tests conducted on the tensile machine, (**a**)—NBR 55 Sh A, (**b**)—NBR 65 Sh A, (**c**)—NBR 75 Sh A.

**Figure 11:**
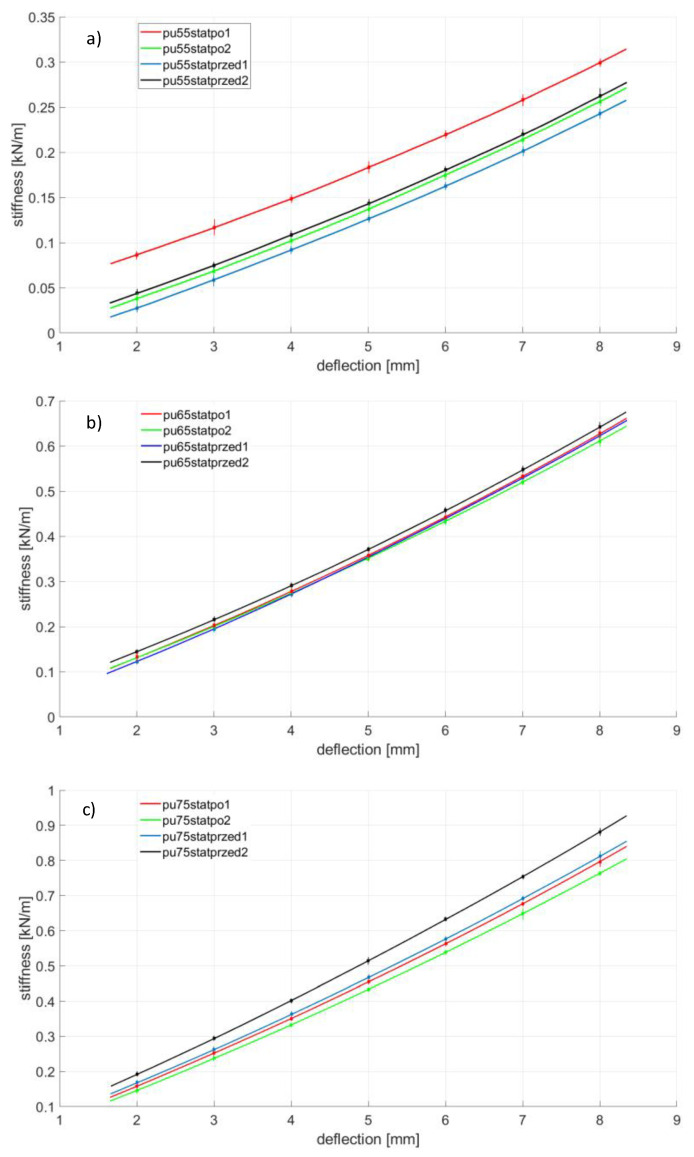
PU changes of stiffness during various tests conducted on the tensile machine, (**a**)—PU 55 Sh A, (**b**)—PU 65 Sh A, (**c**)—PU 75 Sh A.

Rubber ageing is a process consisting of changing the physicochemical and mechanical properties of rubber (related to the degradation of the chemical structure) under the influence of such factors as oxygen, ozone, increased temperature, light radiation, UV radiation, ionizing radiation and chemicals in which the ingredients of the rubber are soluble. The ageing of the rubber can also be significantly accelerated when cyclic stresses are present [18,24]. The first part, Figure 8, Figure 9, Figure 10 and Figure 11, presents the results of strength tests of metal-rubber shock absorbers made of the materials described in Section 2.

In the case of CR rubber with a hardness of 55 ShA, the reduction in stiffness is noticeable in both cases of fatigue tests. It is especially noticeable in the situation where the rubber element was subjected to 100 compression cycles at 50% of its initial length. The situation is similar to the other CR rubber hardness. It should be emphasized here that the measurement of CR 65 with a 50% compression was damaged and was not shown in Figure 8. The initial stiffnesses of shock absorbers made of CR rubber show a great similarity in relation to all tested hardness. However, the similarity of the stiffness of the new samples to the same hardness is not so obvious, as can be seen from the example of EPDM65 (Figure 9) or NBR75 (Figure 10) rubber. Concerning both EPDM and NBR, the tendency of changes in the stiffness of shock absorbers made of them is similar to that in the case of CR rubber, i.e., they decrease their stiffness during the static compression test performed after fatigue actions. This is especially noticeable in the case of fatigue tests in which the rubbers were compressed significantly above the allowable deformation. The situation is slightly different in the case of metal-rubber shock absorbers made of polyurethane (Figure 11). In the case of this material with a hardness of 55 ShA, there was a very small relative reduction in stiffness caused by the fatigue tests carried out, and in one case (PU55), there was even an increase in stiffness. This confirms the strong resistance of this material to long-term fatigue stress.

**Figure 12:**
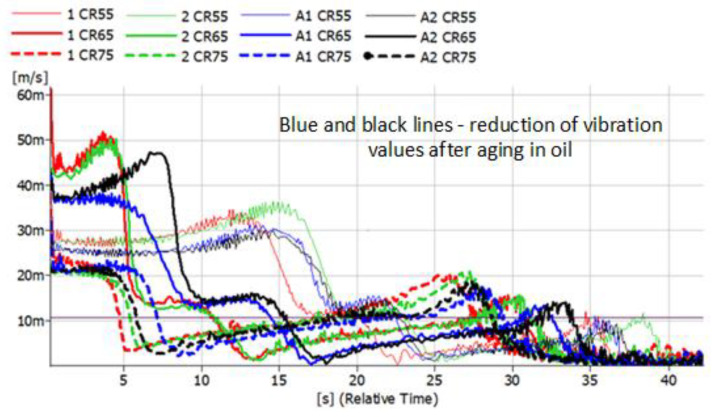
Vibration parameters during the run-down process with CR rubber absorbers installed in the vibration insulation system.

**Figure 13:**
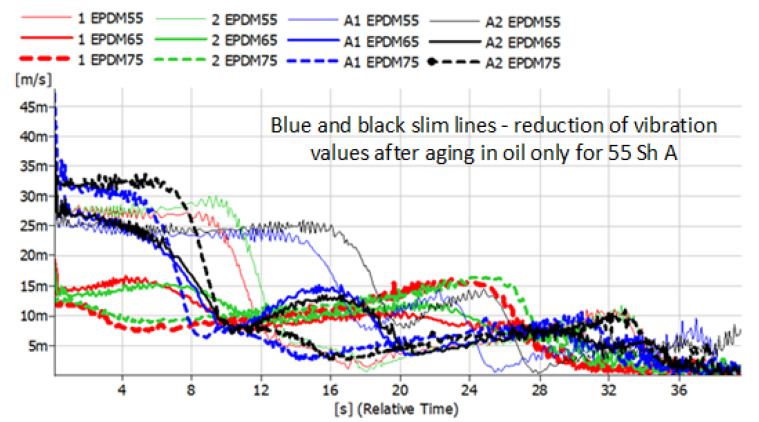
Vibration parameters during the run-down process with EPDM rubber absorbers installed in the vibration insulation system.

**Figure 14:**
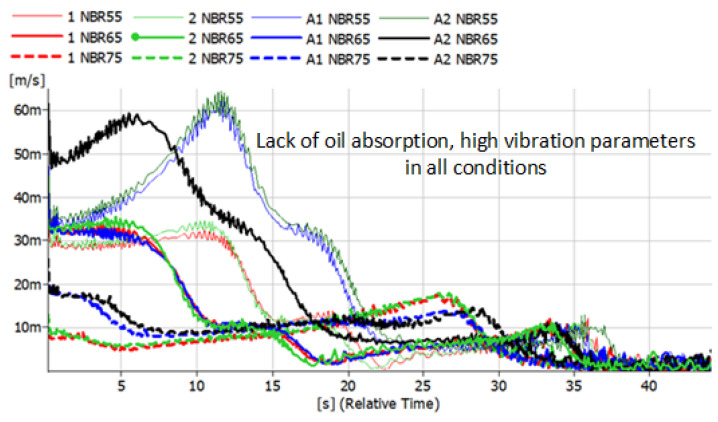
Vibration parameters during the run-down process with NBR rubber absorbers installed in the vibration insulation system.

**Figure 15:**
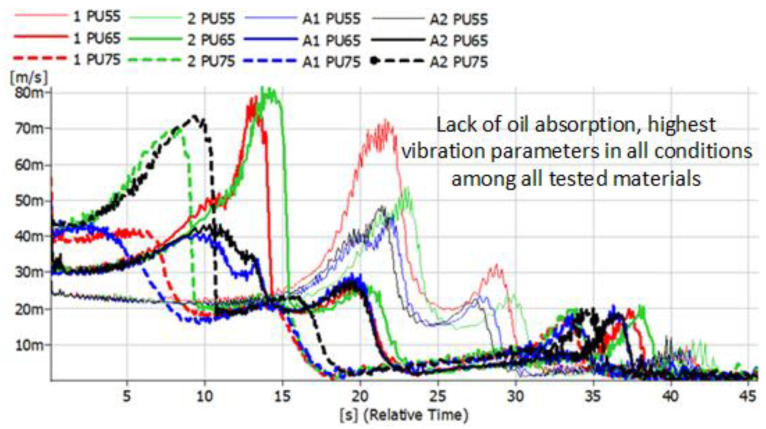
Vibration parameters during the run-down process with PU absorbers installed in the vibration insulation system.

The results presented below refer to metal-rubber shock absorbers made of the same materials as described above. This time, they were tested in conditions similar to real ones and installed as damping elements in the foundations of the rotor machine shown in Figure 5. Vibration accelerations were recorded at four points. However, preliminary analyses allowed us to conclude that the most distinct changes were observed in the VBA point (vertical before absorber). The same tendencies, albeit significantly less pronounced, were visible in the VAA (vertical after absorber) point. There were no significant relationships between HBA and HAA. During the measurements, time courses of vibration accelerations were recorded, which were subjected to integration in the post-processing process, and the spectra of the vibration velocity were presented (Figure 12, Figure 13, Figure 14 and Figure 15).

The convention of marking the individual waveforms of velocities of vibrations during the run-down process adopted in the figures is as follows: red—waveforms recorded for new shock absorbers; green—waveform recorded during the run-down of the machine after 500,000 revolutions; blue—new shock absorbers subjected to the action of petroleum products for 48 h; black—shock absorbers subjected to oil for 48 h and 500,000 forces. The continuous slim line indicates the materials with a hardness of 55 ShA. The continuous light line indicates the waveforms of vibration acceleration specific to the materials with a hardness of 65 ShA. The dashed line indicates the hardness of 75 ShA. Before and after the shock absorbers were subjected to the effects of petroleum products, they were measured and weighed. The results are shown in Table 1.

The tested rotor machine is a supercritical device, which means that it goes through at least one resonant speed during start-up and stop. In Figure 12, Figure 13, Figure 14 and Figure 15, the passage through three renaissance velocities is clearly visible. This is manifested by a sharp increase in amplitude with a decrease in rotational speed (in our case, with the passage of time during the run-down process). The results of the CR rubber obtained at this stage of the research indicate that for all the hardness of this type of rubber, the influence of petroleum products on its vibration damping properties is visible. The possibilities of energy dissipation increased significantly because this type of rubber absorbed the most petroleum products, which can be seen in Table 1. The geometrical dimensions of the shock absorbers made of CR rubber have also changed. Despite improvements of the damping properties, further use of metal-to-rubber shock absorbers made of this material would soon lead to their destruction and, consequently, damage to the amortized machine. In the case of EPDM rubber with a hardness of 55 ShA, a slight decrease in the vibration velocity is observed in the entire range of the tested frequencies; however, already for hardness 65 and 75 ShA, there is an increase in the RMS value of the vibration velocity in the range of higher rotational speeds and a decrease at lower rotational speeds of the rotor. EPDM rubber also absorbs petroleum products, but not as intensely as CR rubber. NBR rubber and polyurethane, during the tests carried out, did not absorb the components of petroleum products in a way that would change their weight or dimensions. The results relating to these two materials are characterized by the highest general level of vibration velocity in the entire measured range, regardless of the state of the rubber elements. In the case of NBR rubber, there is also a correlation between the influence of petroleum products and the hardness of the rubber. NBR55 shows a greater than 50% increase in the value of the vibration velocity amplitudes when passing through the first resonance, while the remaining values of the change are hardly noticeable. The same is the case with polyurethane. With regard to this material, a sharp increase in the value of the vibration velocity is clearly visible when passing through the resonance. The registered amplitude values are the highest among all the tested materials.

## 4. Discussion

Despite the fact that rubber-metal vibration isolators are widely used vibration isolation systems in marine applications, the process of their selection must be carried out very carefully. All factors that may change their characteristics during operation should be taken into account. In relation to devices located in ship power plants, such factors are impulse interactions of considerable value, the source of which are usually underwater explosions and the influence of petroleum products [25]. The conducted tests allow the conclusion that shock absorbers selected in a way that does not take into account these two factors may be destroyed in a very short time, causing deterioration of the dynamic characteristics of the entire drive system.

Particularly important and usually neglected seems to be the influence of exceeding the permissible loads. Most manufacturers of metal-rubber shock absorbers state that the permissible loads during the shock absorber’s operation cannot change its length by more than 10%. However, during the contact of the hull with the shock wave, these values are often exceeded. The obtained results clearly indicate that the phenomena occurring during excessive loads cause permanent changes in the stiffness characteristics of the rubber elements of the shock absorbers. Polyurethane seems to be the most resistant to this phenomenon among the tested materials.

Due to the common occurrence of fuels and oils on board vessels, the selection of shock absorbers should also take into account the resistance of the chosen elastomers to these factors. Rubber manufacturers provide a degree of resistance of individual products to aggressive liquids. However, this information is not present in the carats of shock absorber characteristics. Therefore, vibration isolation engineers for marine propulsion systems must have more knowledge than what is commonly available in catalogues.

## 5. Conclusions

The main aims of this work were verifying vibro-isolator characteristics in the event of a significant overload or impact of petroleum products on them. These goals were achieved by carrying out tests on a tensile testing machine in relation to overload. The characteristics of changes in properties as a result of the impact of petroleum products were achieved by testing the samples exposed to such an impact on a test stand that is a model of the actual foundation system for a rotating machine. The results confirm the existence of a relationship between the two tested extreme effects on most of the tested materials. It seems very important that there is no information on the issues discussed in the article in the literature related to the selection of vibro-isolators, especially in relation to objects exposed to significant impact interactions.

## Figures and Tables

**Table 1:** Basic parameters of the tested absorbers before and after soaking in a mixture of fuel and oil for 48 h.

Material Type	State	Diameter [mm]	Length [mm]	Mass [g]
CR55	Before	19.50	40.07	27.89
	After	19.73	40.56	28.33
CR65	Before	19.49	40.09	28.40
	After	19.55	40.10	28.75
CR75	Before	19.74	40.41	28.97
	After	19.74	40.40	29.05
EPDM55	Before	19.53	39.80	25.95
	After	20.11	41.58	26.90
EPDM65	Before	19.78	40.00	25.95
	After	20.53	41.82	26.73
EPDM75	Before	19.55	40.30	26.44
	After	20.35	41.61	27.75
NBR55	Before	19.32	39.91	25.90
	After	19.43	39.86	25.91
NBR65	Before	19.40	39.88	26.92
	After	19.49	39.88	26.99
NBR75	Before	19.42	40.01	26.60
	After	19.42	39.87	26.57
PU55	Before	19.43	40.14	28.19
	After	19.40	39.89	28.25
PU65	Before	19.41	40.03	28.37
	After	19.42	40.01	28.42
PU75	Before	19.65	40.00	28.63
	After	19.73	40.02	28.66

## Data Availability

The data presented in this study are available on request from the corresponding author. The data are not publicly available due to the fact that they were obtained during research grant which is not finished yet.
